# Increased sugar-sweetened beverage use tendency in pregnancy positively associates with peripartum Edinburgh postpartum depression scores

**DOI:** 10.1038/s41598-021-94790-5

**Published:** 2021-07-28

**Authors:** Chin-Ru Ker, Chen-Hsuan Wu, Chien-Hung Lee, Shih-Han Wang, Te-Fu Chan

**Affiliations:** 1 Department of Obstetrics and Gynecology, Graduate Institute of Medicine, College of Medicine, Kaohsiung Medical University, Kaohsiung, Taiwan; 2 Department of Obstetrics and Gynecology, Kaohsiung Medical University Hospital, Kaohsiung Medical University, 100 Tzyou 1st Road, Kaohsiung, 807 Taiwan; 3 Graduate Institute of Clinical Medical Sciences, Chang Gung University College of Medicine, Taoyuan, Taiwan; 4 Department of Obstetrics and Gynecology, Kaohsiung Chang Gung Memorial Hospital and Chang Gung University College of Medicine, 123 Dapi Road, Niaosong District, Kaohsiung, 833 Taiwan; 5 Department of Public Health, College of Health Science, Kaohsiung Medical University, 100 Shih-Chuan 1st Road, Sanmin District, Kaohsiung, 807 Taiwan; 6 Center of Cancer Research, Kaohsiung Medical University, Kaohsiung, Taiwan

**Keywords:** Human behaviour, Lifestyle modification, Risk factors

## Abstract

The association among sugar sweetened beverages (SSB) consumption, addiction and depression in adults, children and adolescents is widely reported. Dieting patterns during pregnancy is complicated by maternal fetal concerns. Specifically, restrained use of SSB might be potentially a source of perinatal distress. The current study modified diagnostic criteria for Substance Use Disorder (SUD) in Diagnostic and Statistical Manual of Mental Disorders, Fifth Edition (DSM-5), into SSB-specific questions to assess SSB use tendency. Edinburgh Postpartum Depression Scores (EPDS) is used to assess maternal distress during pregnancy. One hundred and ninety-six consecutive pregnant women receiving antenatal care at Kaohsiung Medical University Hospital were invited to participate in this study. In the first trimester, 46.6% of women had none or 1 DSM-5 symptom, 27.0% had 2–3 symptoms, while 26.4% had ≥ 4 symptoms. The mean numbers of DSM-5 symptoms in each trimester were found to be 2.5 ± 2.25, 2.6 ± 2.45, 2.4 ± 2.43 for the first, second and third trimester, respectively, *p* = 0.750. While EPDS score showed no difference among DSM-5 symptoms 0–1, 2–3 and ≥ 4 groups in the first trimester (8.1 ± 4.59, 8.4 ± 5.00, 8.8 ± 4.82, *p* = 0.343), women with ≥ 4 DSM-symptoms was found significantly higher EPDS scores than those with < 4 DSM-symptoms in the second (7.2 ± 4.81, 7.7 ± 4.98, 8.8 ± 4.33, *p* = 0.030) and third trimester (6.8 ± 5.00, 7.2 ± 4.63, 8.7 ± 5.24, *p* = 0.019). The relationship remained significant after adjusting for covariates including actual SSB amount consumed (adjusted β = 0.25 with 95% confident interval (CI) 0.04–0.45 and 0.21 with 95% CI 0.04–0.38 for the second and third trimesters, respectively). Overall, the study is the first to characterize the positive relationship between SSB use tendency and antenatal distress in pregnancy, independent of actual SSB amount consumed. The observational nature of the study design precludes inferences of its underlying socio-psychomotor mechanisms, although restrained SSB use in pregnancy is suspected to contribute. The novel employment of modified SSB-specific DSM-5 scores and EPDS in this setting is feasible and further validation is promising. With better understanding and awareness, pregnant women with increased SSB use tendency should be properly counseled with special attention to their mental state.

## Introduction

Pregnancy is stressful for women both physically and emotionally. As high as over half of pregnant women report anxiety or depression at some points in their pregnancy course^1^. Major obstetric practice guidelines worldwide are in line with calling for practitioners’ attention on this issue, including the American College of Obstetricians and Gynecologists^2^, National Institute for Health and Care Excellence (NICE) in the United Kingdom^3^ and Centre of Perinatal Excellence (COPE) in Australia^4^. The incidence of peripartum depression is 10–15% with considerable underestimation^5–9^. Symptoms of depression are often unspecific, coinciding with normal peripartum physiology like changes in sleep, appetite and libido. Preoccupation with childbearing makes women less aware of their changes in mood. Stigma associated with mental illness also prevents women from seeking help^9^. Less than 20% of women in whom postpartum depression was diagnosed reported their symptoms to a health care provider^8,10,11^. These are all barriers leaving much room for improved sensitivity and care strategies for the affected women.

Sugar-sweetened beverages (SSB) consumption is known to associate with increased risk for depression. The link was first reported in the children and adolescent population^12^. It is later generalized to the adult populations^13–16^. For example, Knuppel et al. found a 23% increased odd for depression for men with higher intake of SSB, independent of health behaviors, socio-demographic and diet-related factors in their cross-sectional and 5-year follow-up study^14^. Hu and authors concluded a 25% higher risk of depression with ingestion of 3 cans of cola per day in their meta-analysis of observational studies^13^. SSB consumption is also known to produce addictive effects (including craving, tolerance and withdrawal) in both animal and human studies^17–19^. Withdrawal from sugar causes dopamine deficiency, leading to depression, decreased performance, attention deficit, hyperactivity and distraction^17,20^. These symptoms were found to be relieved upon sugar fixes. The investigation of SSB use pattern in pregnant women is particularly intriguing in that women are motivated to pursue healthier diet to optimize pregnancy outcomes for both herself and her baby^21,22^. Skreden et al. reported that Norwegian women largely replaced their alcohol and caffeinated drinks with water, fruit juice and milk during pregnancy^21^. The impact of SSB use tendency and women’s mental health during pregnancy has not been characterized before. Additionally, whether restraining from SSB use itself causes psychological distress is not known. The interplay among SSB use tendency, SSB amount consumption and psychological distress in pregnancy is warranted to be the subject of investigation.

**Figure 1:**
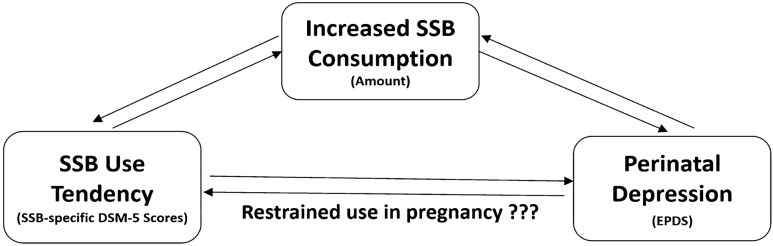
Hypothesized relationships among SSB use tendency, SSB intake amount and perinatal depression.

Addiction-like eating behaviors parallel substance use disorders (SUD) in many ways: craving, failure to fulfill major role obligations, social or interpersonal problems, and use in physically hazardous situations^23^. Meule et al. thought likewise and proposed applying DSM-5 SUD diagnostic criteria to describe overeating^24^. In the same light, we borrowed the strengths in DSM-5 SUD criteria, for its wide and easy application, international recognition and validation in numerous research literatures. To embody and quantify SSB use tendency, we modified the diagnostic criteria for substance use disorder (SUD) of the fifth revision of the Diagnostic and Statistical Manual of Mental Disorders (DSM-5) to become SSB-specific survey questions. We hypothesize that SSB use tendency measured by SSB-specific DSM-5 items during pregnancy is associated with perinatal depression (Fig. 1).

## Material and methods

This longitudinal observational cohort study was conducted at Kaohsiung Medical University Hospital (KMUH). One hundred and ninety-six consecutive pregnant women aged 20 and above were recruited in their regular antenatal care visits at KMUH. A structured 20-min questionnaire was given to them in their first, second and third trimester of pregnancy. Written informed consent with full explanation about the purpose and method of the study were obtained from all targeted women. Those who refused to participate, were incomprehensible to Mandarin Chinese, failed to complete all 3 questionnaires, experienced termination of pregnancy, early intrauterine fetal demise or discontinued their antenatal care at KMUH were excluded. A total of 163 participants’ responses were collected for analysis. The drop-out rate was 16.8%. The project was approved by the hospital Institutional Review Board (KMUHIRB-SV(I)-20160062) and conducted in accordance with the Declaration of Helsinki.

In the five-section questionnaire, the participant’s demographic characteristics, comorbidities, obstetric history, family history, SSB consumption patterns and level of emotional distress were assessed. We defined women who consumed one or more servings of any type of SSB per week over the previous month as SSB consumers^25,26^. The characterization of SSB consumption in terms of amount, type, frequency and level of dependence was examined in detail. Commonly consumed SSBs, including sweetened teas, soft drinks, sports drinks and fruit drinks available in Taiwan were listed. The amount consumed was estimated using product labels or visualized analogue when labels were not available. The frequency of use was calculated with occurrences per day and days per week. The participant’s psychological stress or distress for the previous 7 days was measured using Edinburg postnatal distress scale (EPDS), which was well studied in previous studies^27–32^ and validated in Mandarin Chinese^33,34^. To describe participants’ SSB consumption behaviors, DSM-5 diagnostic criteria for substance use disorder were modified to measure specific symptoms for SSB use (Table 1). The main goal was to describe the psychological tendency of SSB use, rather than to measure the actual SSB consumed. A validity test for various signs of tendencies developed was not available. Alternatively, the number of positive responses to symptoms described was counted for each participant. The pregnant women with more symptoms reported were considered having higher psychological tendencies of SSB use.

**Table 1:** Modified DSM-5 diagnostic criteria for SSB use tendency.

	Questions	No	Yes^a^
1	Did you consume more amount of SSB than you intended to or more often than you used to?		
2	Did you try in vain to stop drinking SSB or still have strong desire to drink?		
3	Did you always drink SSB while working or spend excessive time searching for SSB at leisure time?		
4	Did you always have strong desire for SSB that you cannot quit until drinking it?		
5	Is your work performance affected by SSB consumption?		
6	Did you continue to drink SSB even if it harms your relationship with family or friends?		
7	Did you abandon or reduce time spent on your friends or other hobbies because of SSB use?		
8	Did you continue to drink despite of body weight gain due to SSB use?		
9	Would you continue to drink even if you know it might harm your health?		
10a	Compared to SSB used in the past, did you have to acquire more SSB to reach the same level of satisfaction?		
10b	If the same amount of SSB was consumed as in the past, would you feel unsatisfied?		
11a	Would you feel uneasy, angry or uncomfortable when SSB is forbidden or reduced in amount?		
11b	When the ban was reversed, would it relieve your uneasiness, anger or discomfort?		

^a^Each positive response scores 1 point.

Data analyses were performed using survey modules of Stata v13.0 (StatCorp, College Station, TX, USA). Proportions and means (± standard deviation) were employed to describe the distribution of demographic characteristics for the pregnant cohort. To reduce residual confounding effect, all multivariable models were adjusted for age, educational level, body mass index, nulliparity, and comorbidity. With dummy variables representing categorical variables, we used multiple regression models to evaluate the difference in the amount of SSB consumed and the number of SSB-associated DSM-5 symptoms among the 3 trimesters. Tests for linear trends in the amount of SSB intake and depression score across increasing groups of DSM-5 symptoms (i.e., 0–1, 2–3, and ≥ 4) were performed using multiple linear regression models by categorizing DSM-5 symptoms and treating scored variable as continuous. Partial regression coefficients of DSM-5 symptom number regressed on EPDS score were obtained from multiple linear regression models adjusted for covariates. To evaluate the scrolling effects of risk factors on EPDS score during the 3 trimesters, data were sequentially assessed according to 3 follow-up period (first, first + second, and first + second + third trimesters). We used generalized estimating equations with autoregressive correlation structure to assess data over first + second, and first + second + third trimesters of follow-up. P-values less than 0.05 indicate statistical significance.

### Ethical approval and informed consent

Ethics approval by the Institutional Review Board of Kaohsiung Medical University Hospital had been obtained for data analysis.

## Results

The study is a single-centered observational study with high response rate (83.2%). This is a cohort study with repeated measurements (3 pregnant trimesters) for each pregnant woman. The statistical power was 96.86% (with alpha = 0.05); 163 participants were repeatedly measured for 3 times, with squared partial correlation = 0.0291, and 6 controlled covariates. The power is over 80%.

### Demographic characteristics

Demographic characteristics of these participants were presented in Table 2. The mean age of the cohort was 33.9 ± 4.6 years old; pre-pregnancy mean body mass index computed to be 22.7 ± 3.6 kg/cm^2^. The weight was measured in kilograms and height in centimeters on an ultrasonic electronic weight and height scale in each trimester. Fifty-three point four percent of the studied group was nulliparous. Thirty-five percent of them reported comorbidities, including 8 hypertension, 9 overt diabetes, 14 thyroid diseases, 6 autoimmune disorders and 7 cardiovascular diseases. The highest levels of education attended were 14.1% high school, 71.2% college and 14.7% graduate school. Among the participants, 19.0% (31/163) reported no regular SSB intake, compared to 81.0% (132/163) with regular SSB use. The numbers of participants using alcohol (n = 3, 1.84%) and tobacco (n = 4, 2.45%) were minimal in this cohort.

**Table 2:** Demographic characteristics of the studied cohort.

Variables	Sugar-sweetened beverage intake		Total^a^
	No^a^	Yes^a^	
Number	31	132	163
Age, years	35.4 ± 4.8	33.5 ± 4.5	33.9 ± 4.6
**Education level**			
High school	12.9%	14.4%	14.1%
College	71.0%	71.2%	71.2%
Graduate school	16.1%	14.4%	14.7%
Weight, Kilograms	57.6 ± 9.6	58.5 ± 10.3	58.4 ± 10.2
Height, centimeters	160.4 ± 4.6	160.3 ± 5.3	160.4 ± 5.2
BMI^b^, Kg/cm^2^	22.3 ± 3.4	22.7 ± 3.6	22.7 ± 3.6
**Nulliparity**			
No	48.4%	46.2%	46.6%
Yes	51.6%	53.8%	53.4%
**Comorbidity**			
No	58.1%	66.7%	65.0%
Yes	41.9%	33.3%	35.0%

^a^Data are given in number, percentage or mean ± standard deviation.

^b^BMI at study entry.

### SSB use in each trimester

SSB intake behavior and numbers of positive DSM-5 symptoms in each trimester were presented in Table 3. Percentage of women claimed to have SSB regular intake was 81.0%, 81.6% and 84.7%; corresponding to mean SSB amount consumed of 545.1 ± 209.77 mL/day, 553.9 ± 250.27 mL/day and 563.8 ± 275.11 mL/day in the first, second and trimester, respectively. In the first trimester, 46.6% of women had ≤ 1 DSM-5 symptom, 27.0% had 2–3 symptoms, while 26.4% had ≥ 4 symptoms. In the second trimester, 49.1% of women had ≤ 1 DSM-5 symptom, 27.6% had 2–3 symptoms, while 23.3% had ≥ 4 symptoms. In the third trimester, 53.4% of women had ≤ 1 DSM-5 symptom, 20.3% had 2–3 symptoms, while 26.4% had ≥ 4 symptoms. The mean numbers of DSM-5 symptoms in each trimester did not reach statistical significance (2.5 ± 2.25, 2.6 ± 2.45, 2.4 ± 2.43 symptoms for the first, second and third trimesters, respectively, *p* = 0.750).

**Table 3:** Sugar-sweetened beverage (SSB) intake and DSM-5 symptoms in each trimester of pregnancy.

	Trimesters			*p* value^a^
	First (n = 163)	Second (n = 163)	Third (n = 163)	
**SSB intake**				
No	19.0%	18.4%	15.3%	0.701
Yes	81.0%	81.6%	84.7%	
Mean amount of intake, mL/day^2^	545.1 ± 209.77	553.8 ± 250.27	563.8 ± 275.11	0.777
Median (interquartile range)	500 (350, 750)	500 (350, 750)	500 (350, 750)	
**No. of SSB *DSM-5* symptoms**				
0–1 symptom	46.6%	49.1%	53.4%	
2–3	27.0%	27.6%	20.3%	
≥ 4	26.4%	23.3%	26.4%	
Mean no. of symptoms^b^	2.5 ± 2.25	2.6 ± 2.45	2.4 ± 2.43	0.750

^a^*p* values for the differences across 3 pregnant trimesters, adjusted for age, educational level, body mass index, nulliparous, and comorbidity in pregnancy.

^b^Data are given in mean ± standard deviation.

### Association between SSB-related DSM-5 scores and EPDS

The number of DSM-5 symptoms in each trimester was then correlated to the amount of SSB use and EPDS scores. The results were given in Table 4. For all three trimesters, a significant increase of SSB intake amount was found to be associated with increased number of DSM-5 symptoms. In the first trimester, mean SSB intake amount consumed was 330.3 ± 250.21 mL/day for responders with ≤ 1 DSM symptom; 452.3 ± 288.93 mL/day for those with 2–3 symptoms; and 626.7 ± 244.79 mL/day for ≥ 4 symptoms (*p* < 0.001). Similarly, in the second trimester and the third trimester, mean SSB intake amount consumed was increased along with more DSM symptoms.

**Table 4:** Sugar-sweetened beverage (SSB) intake amounts and depression scores among women with different number of DSM-5 symptoms in each trimester of pregnancy.

	Trimesters (n = 163)		
	First	Second	Third
**Amount of SSB intake, mL/day**^**a**^			
No. of SSB *DSM*-5 symptoms			
0–1	330.3 ± 250.21	295.0 ± 247.08	389.1 ± 286.36
2–3	452.3 ± 288.93	518.9 ± 245.71	500.0 ± 307.46
≥ 4	626.7 ± 244.79	702.6 ± 317.71	638.4 ± 352.18
* P* for linear trend^b^	< 0.001	< 0.001	0.001
**Depression score**^**a**^			
No. of SSB *DSM*-5 symptoms			
0–1	8.1 ± 4.59	7.2 ± 4.81	6.8 ± 5.00
2–3	8.4 ± 5.00	7.7 ± 4.98	7.2 ± 4.63
≥ 4	8.8 ± 4.82	8.8 ± 4.33	8.7 ± 5.24
*P* for linear trend^b^	0.343	0.030	0.019

^a^Data are presented as mean ± standard deviation.

^b^*p* values for linear trend were obtained using regression models adjusted for age, educational level, body mass index, nulliparous, and comorbidity in pregnancy.

EPDS score showed no difference among number of DSM-5 symptoms ≤ 1, 2–3 and ≥ 4 groups in the first trimester (8.1 ± 4.59, 8.4 ± 5.00, 8.8 ± 4.82, *p* = 0.343). However, women with ≥ 4 DSM-symptoms was found significantly higher EPDS scores than those with < 4 DSM-symptoms in the second (7.2 ± 4.81, 7.7 ± 4.98, 8.8 ± 4.33, *p* = 0.030) and third trimesters (6.8 ± 5.00, 7.2 ± 4.63, 8.7 ± 5.24, *p* = 0.019). Moreover, women with ≤ 3 DSM-5 symptoms had gradual decrease of EPDS scores after the second trimester. The pattern is not observed in those reporting ≥ 4 DSM-5 symptoms, who had maintained stable EDPS scores throughout the pregnancy, leading to a statistically significant difference after the second trimester.

### Risk factors adjusted for increased EPDS scores

After adjusting for covariates, pregnant women with a higher educational level were found to have lower EPDS score in the 3 pregnant periods studied (Table 5). Pregnant women with every increase in body mass index were associated with 0.17 (95% CI: − 0.31, − 0.03) and 0.14 (95% CI: − 0.25, − 0.03) decreased EDPS scores during the first + second trimesters and first + second + third trimesters, respectively. Moreover, each increase in DSM-5 symptoms of SSB use was associated with an increase of 0.25 (95% CI: 0.04, 0.45) and 0.21 (0.25, 0.38) EPDS scores during the first + second trimesters and first + second + third trimesters, respectively.

**Table 5:** The adjusted mean differences of depression scores during pregnancy periods associated with DSM-5 symptoms of sugar-sweetened beverage (SSB) intake and potential risk factors.

	Depression scores in pregnancy trimesters					
	First		First + second		First + second + third	
	Adj. β^a^	(95% CI)^a^	Adj. β^a^	(95% CI)^a^	Adj. β^a^	(95% CI)^a^
**Continuous variables**						
Age, year	0.08	(− 0.09, 0.25)	0.13	(− 0.02, 0.28)	0.10	(− 0.05, 0.24)
Body mass index, Kg/m^2^	− 0.03	(− 0.25, 0.19)	− 0.17*	(− 0.31, − 0.03)	− 0.14*	(− 0.25, − 0.03)
*DSM*-5 symptoms, no	0.31	(− 0.06, 0.69)	0.25*	(0.04, 0.45)	0.21*	(0.04, 0.38)
**Categorical variables**						
Educational level						
High school	Ref		Ref		Ref	
College	− 3.03*	(− 5.31, − 0.75)	− 2.76*	(− 4.81, − 0.72)	− 2.73*	(− 4.70, − 0.77)
Graduate school	− 3.86*	(− 6.69, − 1.02)	− 3.06*	(− 5.62, − 0.50)	− 2.62*	(− 5.09, − 0.14)
Amount of SSB intake, mL/day						
Non-intake	Ref		Ref		Ref	
1–500	− 1.69	(− 3.68, 0.32)	− 0.18	(− 1.32, 0.95)	0.24	(− 0.65, 1.13)
> 500	− 1.65	(− 4.01, 0.71)	0.16	(− 1.17, 1.50)	0.41	(− 0.62, 1.44)
Nulliparous						
No	Ref		Ref		Ref	
Yes	0.67	(− 0.89, 2.23)	0.16	(− 1.23, 1.55)	0.52	(− 0.82, 1.86)
Comorbidity						
No	Ref		Ref		Ref	
Yes	0.50	(− 1.09, 2.10)	0.56	(− 0.87, 2.00)	0.44	(− 0.95, 1.82)

^a^Adjusted regression coefficients (Adj. β) were adjusted for all covariates listed in the table. The 95% confidence interval (CI) were calculated for the Adj. β

*Denotes *p* value < 0.05.

## Discussion

The current study provides the first and novel evidence that SSB use tendency in pregnancy is linked to perinatal distress. It has not been discussed in the obstetric settings. In the past, reports on SSB use in pregnancy mainly centered around its negative physical effects in both the mother and the fetuses: maternal metabolic syndrome, obesity, macrosomia, congenital heart diseases and infant cognition impairment are some to exemplify^21,22,35–37^. Here we assert that ignorant or unrestrained SSB use in pregnancy should illicit caution, for it might signal distressed mental state. SSB is low-cost, easily accessible, and seemingly much less guilty to consume during pregnancy in Taiwan^38–40^. Pregnant women easily rely on SSB as a coping strategy to her distress and even become addicted unknowingly. This might in turn become a source of stress itself when the level of satisfaction is not met due to tolerance or withdrawal (Fig. 1).

A higher SSB use tendency was found to positively relate to depression scores, with the trend most pronounced after the second trimester (Table 4). The trend remained significant after adjusting covariates (Table 5). The observational nature of the study precluded making inferences for the underlying biopsychosocial mechanisms, although some theories could be lent from relevant literatures targeting the general population (Fig. 1). Studies that proposed SSB consumption could lead to higher risk of depression were readily found^12–17^. Other scholars had reversed reasoning and suggested depressed people preferentially consumed more sweets^41–44^. Yet others embraced the idea that the influence was mutual and bidirectional^45^. The applicability of these theories to SSB use tendency in distressed pregnant women awaited further studies with better designs that could provide higher-leveled evidence.

Compared to the general population, eating behaviors and food choices during pregnancy can be further perplexed with maternal fetal concerns. Motivated women take pregnancy as a window of opportunity for healthy lifestyles, and therefore restrain themselves from unhealthy dieting. To illustrate, Skreden and colleagues provided a classic descriptive study that demonstrated how Norwegian women replaced their alcohol, coffee, sugar-sweetened beverages and artificially sweetened beverages with water, fruit juice and milk from pre-pregnancy to early pregnancy in a statistically significant manner^21^. External instructions urging reduced SSB use in pregnancy are also common^22,37,46,47^. In this respect, retraining SSB use in pregnant women already with high SSB use tendency is likely a stressor itself, regardless of SSB amount consumed (Fig. 1). Our data supports the hypothesis by demonstrating persistent positive association between depression scores and SSB-specific DSM-5 symptoms after adjusting for SSB intake amount (Table 5). Although the relationship between restrained dieting and depression is well discussed in the general population^43,48^, its relevance in the obstetrics and SSB requires further investigational trials to prove.

We employed DSM-5 diagnostic symptoms for substance use disorder to measure psychological tendency of SSB consumption. The DSM-5 criteria for substance use disorder is a recently established process to measure substance use disorder, including alcohol and tobacco use^49–51^, and this method has been well applied to measure betel-quid (one commonly consumed substance in Taiwan) use disorder^52^. Also interesting is the observation that participants with greater SSB use tendency (≥ 4 symptoms) maintained high EPDS throughout pregnancy, in contrast to those with less tendency having gradually decreased EDPS later in gestation (Table 4). SSB-specific DSM-5 score of 4 could therefore serve as a threshold for increased risk for SSB use tendency and perinatal distress based on our observation.

Edinburgh Postnatal Depression Scale (EPDS) was utilized in the current study for maternal stress stratification throughout pregnancy. The aim was not to diagnose depression, rather to depict the differential levels of distress experienced by the study cohort. The higher scores reported by the participants signifies higher level of distress. The application of EPDS in the antenatal settings is well validated in previous studies^53–62^, some extending across all 3 trimesters during pregnancy^28,61^. Bergink and colleagues particularly proposed repeated screening with EPDS in each trimester to better detect women at risk for depressive symptoms^61^. Khanlari et al. further urged that EDS should be conceptualized as a spectrum and its scores interpreted more dynamically^29^. We embraced the insightful views and believed the application of EPDS in the current study justified.

There are several limitations to address. The hospital is a tertiary teaching center, complying with national health insurance that covers patients from all socioeconomic backgrounds. The potential for sample capture bias is minimal with indifferent sequential participant invitations. But the cohort has higher proportions of advanced maternal age and high-risk pregnancies, who are prone to increased anxiety and mental distress. These factors do not interact with our major findings after statistical adjustment. Other common substances (e.g., alcohol and tobacco) were considered. The positive users were so few (1.84% alcohol users and 2.45% tobacco users) that their effects were minimal. The observational nature precludes validated explanations for the underlying mechanisms leading to this phenomenon but opens further investigation opportunities for clarification. Detailed interrogation to patients’ motives of SSB use or interventional studies could be designed in future studies. The study interest lies specifically to SSB use pattern with pending validation for the utilization of DSM-5 SUD criteria modified for SSB. Total amount of added sugars derived from other sources of food was not accounted for.

## Conclusion

A positive relationship between SSB use tendency in pregnancy and maternal mental distress level was found in the current study. The relationship held true after adjusting for covariates and actual SSB amount consumed. SSB impacts maternal mental distress not just by physically the amount taken or the physiological effect of carbohydrate, but also through a psychomotor mechanism that involves craving, withdrawal and tolerance. SSB-specific DSM-5 scores and EPDS are user friendly tools that help decipher the relationship between SSB use tendency and maternal mental distress levels. With better understanding and awareness, pregnant women with increased SSB use tendency should be properly counseled and followed both physically and mentally.

## Data Availability

The datasets analyzed during the current study are available from the corresponding author on reasonable request.
